# 

**Published:** 1873-12

**Authors:** 


### CONTENTS:

### ORIGINAL COMMUNICATIONS.

ARTICLE I.—Chemistry of the Oral Secretions and Their Action on the Teeth. By Edward C. Chase........ 33

ARTICLE II.—Dental Pathology. By W. L. Younger, M. D........ 34

ARTICLE III.—Microscopy. By S. P. Cutler, M. D., D. D. S........ 34

ARTICLE IV.—The American Academy of Dental Science. By E. N. Harris, D. D. S........ 35

ARTICLE V.—Cleaning and Smoothing Palatine Surfaces of Rubber Plates. By B. M. Wilkerson, D. D. S., M. D........ 35

### SELECTED ARTICLES.

ARTICLE VI.—Diseases of the Antrum. By A. Van Best, M. D., F. R. C. S........ 35

ARTICLE VII.—Pathological Dentition. By James W. White, M. D........ 35

ARTICLE VIII.—Case of Facial Paralysis. By Gouverneur M. Smith, M. D........ 36

ARTICLE IX.—Laws of Transmission of Resemblance from Parents to Children. By John Stocktonhough, M. D........ 36

ARTICLE X.—Hereditary Syphilis........ 36

ARTICLE XI.—Carbolic Acid as an Anæsthetic. By Andrew H. Smith, M. D........ 37

ARTICLE XII.—The Theory of Counter Irritation........ 37

### EDITORIAL, ETC.

The Birthplace of Modern Dentistry........ 37

The Irritation of Rubber Plates........ 37

New Means of Relieving Tooth Ache........ 37

Corrections........ 37

### MONTHLY SUMMARY.

Tempering Steel........ 37

Removing a Foreign Body from the Nose........ 37

Treatment of Chilblains........ 37

Nasal Calculus........ 37

Female Professional Education........ 37

Deep Injection of Chloroform in Tic Douloureux........ 38

Atropia and Salivation........ 38

The Restoration of Chloroform........ 38

A Dentist fined for giving Chloroform........ 38

A Pre-Historic Skull........ 38

Chloral for Toothache........ 38

### BIBLIOGRAPHICAL.

Practical Histology in Vienna, and the Microscopical Stndy of Blood and Epithelium........ 38

Circulars of Information of Bureau of Education........ 38

Mechanism of the Ossicles of Hearing and the Membrane of the Round Window........ 38

Transactions of California State Dental Association........ 38

The Sanitarian........ 38

The Herald of Health,........ 38

The American Agriculturist........ 38

The Physician's Visiting List for 1874........ 38

Wood's Household Magazine........ 38

Vick's Floral Guide for 1874........ 38

